# Induction of Protective Genes Leads to Islet Survival and Function

**DOI:** 10.1155/2011/141898

**Published:** 2011-12-14

**Authors:** Hongjun Wang, Christiane Ferran, Chiara Attanasio, Fulvio Calise, Leo E. Otterbein

**Affiliations:** ^1^Division of General Surgery, Department of Surgery, Medical University of South Carolina, Charleston, SC 29425, USA; ^2^Department of Surgery, Beth Israel Deaconess Medical Center, Harvard Medical School, Boston, MA 02215, USA; ^3^Center of Biotechnologies, Cardarelli Hospital, 80131 Napoli, Italy

## Abstract

Islet transplantation is the most valid approach to the treatment of type 1 diabetes. However, the function of transplanted islets is often compromised since a large number of **β** cells undergo apoptosis induced by stress and the immune rejection response elicited by the recipient after transplantation. Conventional treatment for islet transplantation is to administer immunosuppressive drugs to the recipient to suppress the immune rejection response mounted against transplanted islets. Induction of protective genes in the recipient (e.g., heme oxygenase-1 (HO-1), A20/tumor necrosis factor alpha inducible protein3 (tnfaip3), biliverdin reductase (BVR), Bcl2, and others) or administration of one or more of the products of HO-1 to the donor, the islets themselves, and/or the recipient offers an alternative or synergistic approach to improve islet graft survival and function. In this perspective, we summarize studies describing the protective effects of these genes on islet survival and function in rodent allogeneic and xenogeneic transplantation models and the prevention of onset of diabetes, with emphasis on HO-1, A20, and BVR. Such approaches are also appealing to islet autotransplantation in patients with chronic pancreatitis after total pancreatectomy, a procedure that currently only leads to 1/3 of transplanted patients being diabetes-free.

## 1. Introduction

Type 1 diabetes (T1D) is caused by the death of insulin-producing pancreatic *β* cells within the pancreas. Islet transplantation, a procedure that can restore the body's blood glucose level in a physiological manner, holds the most promise in treating patients with T1D [1]. With the success of the Edmonton protocol, clinical islet transplantation can provide T1D patients with sustained and improved glycemic control and a period of insulin independence [2]. There are, however, many problems with this procedure. First, nonimmune-related stress during islet isolation and transplantation results in a significant number of islets undergoing apoptosis immediately after transplantation. Thus, at least 2-3 donors are needed per recipient to ensure survival of a sufficient islet cell mass to achieve insulin independence [3–6]. Second, those islets that survive need to sustain an allograft rejection response and recurrence of autoimmunity mediated by the recipients' T cells, natural killer cells, monocytes, and cytokines, otherwise additional islet/*β* cell death would ensue [7]. Both obstacles have significantly limited clinical application of islet transplantation for the treatment of T1D. Similarly, the effectiveness of autologous islet transplantation, a procedure currently implemented in the clinic to treat patients suffering from chronic pancreatitis, is also impacted by *β* cell apoptosis posttransplantation, that is, only 1/3 of the patients are insulin-free after total pancreatectomy and islet autotransplantation [8–10]. Donor islet quality plays a critical role in determining the outcome of allo- and autotransplantation of islet grafts, with stress-induced *β* cell apoptosis greatly contributing to failure of these procedures. Thus, novel strategies that enable *β* cell resistance to stress would prevent *β* cell apoptosis and reduce or even eliminate immune rejection and recurrent autoimmunity thereby benefiting clinical application of islet transplantation.

The etiology of T1D is complex and poorly understood. Many factors including genetic susceptibility, environmental factors, the immune system, and *β* cells themselves were found to participate in the pathogenic process of this disorder [11]. A variety of pathogenic pathways including CD8^+^ cytolytic T-cell-mediated killing, cytokine exposure, apoptosis caused by fatty acid synthase and fatty acid synthase ligand can lead to immune-mediated destruction of *β* cells during the onset of T1D [12], suggesting that individual therapeutic strategies targeting one pathway may not be sufficient to cure T1D [13, 14].

**Figure 1:**
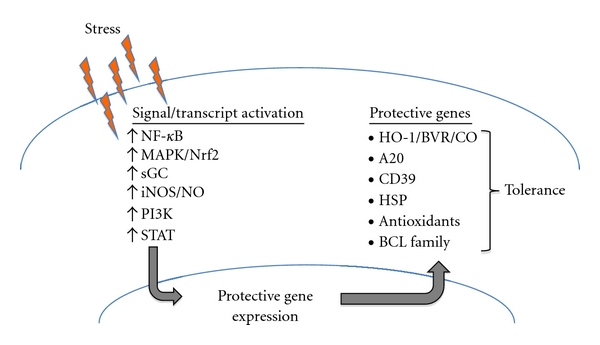


A protective gene is a gene that is upregulated in response to stress through specific signaling cascades and transcription factor regulation that when induced participate in promoting cell survival [15] (Figure 1). Many protective genes including HO-1, A20, B-cell lymphoma 2 (Bcl-2), Bcl-x, heat shock proteins, biliverdin reductase (BVR), and antioxidant enzymes have been found to be expressed in pancreatic islets, and their expression leads to protection against apoptosis and other injuries while their absence leads to a heightened response to stress or in the case of HO-1, low fecundity, and a shortened lifespan fraught with continuous inflammatory sequelae throughout life [16], and in the case of A20 unfettered inflammation and death within 3–6 weeks of birth [17].

## 2. HO-1

HO-1 is the rate-limiting enzyme that degrades heme to generate equal molar amounts of carbon monoxide (CO), biliverdin, and iron [18]. Biliverdin is rapidly converted into bilirubin by biliverdin reductase, and iron is sequestered into ferritin. HO-1 is a ubiquitous stress protein and can be induced in many cell types by various stimuli [19]. There is increasing evidence indicating that induction of HO-1 provides cellular protection against transplant rejection [20, 21], hypertension [22], hyperoxia [23], acute pleurisy [24], ischemia reperfusion injury [25], and endotoxic shock [22]. HO-1 is intimately involved in the inflammatory, apoptotic, and proliferative properties of the cell in response to a given stress. The anti-inflammatory properties ascribed to HO-1 are an important means of protection and survival. Mice deficient in HO-1 develop a chronic inflammatory state that progresses with age. The first HO-1 deficient human died of an inflammatory syndrome at the age of six [26, 27]. Another case of human HO-1 deficiency was reported recently in a young girl with congenital asplenia, who presented with multiple organ dysfunction as well as hemolysis, inflammation, nephritis, and resistance to therapy [28]. There is evidence that each product of HO-1, biliverdin/bilirubin, CO, or ferritin accounts for its protective effects both when used alone or in combination [29–32]. 

## 3. HO-1 Increases Survival and Function of Islet Allograft

More than half of the islet tissue is lost in both the syngeneic and the autoimmune transplantation settings at 2-3 days posttransplantation, which contributed to the primary nonfunction of transplanted islets [33]. Considering the shortage of islet donors, prevention of *β* cell apoptosis will effectively reduce the number of donors required for each transplant and increase the success rate for this procedure. Early islet/*β* cell apoptosis after transplantation is typically induced by nonimmune-mediated stressors including prolonged hypoxia during the revascularization process, nutrition deprivation, ischemia reperfusion injuries, and proinflammatory and cytokine expression [5]. HO-1 expression is observed in islets under stress conditions, such as during islet isolation prior to transplantation or cytokine treatment with IL-1*β* and IFN*γ* [6]. Induction of HO-1 pharmacologically or via gene transfer protects islets from stress-induced apoptosis in both the *in vitro* and the *in vivo* settings. *In vitro*, several studies have showed that HO-1 induction in *β* cell lines, primary murine, or human islets protects against apoptosis induced by TNF-*α* and cyclohexamide (CHX), interleukin-1*β* (IL-1*β*), and Fas [34–36]. Transduction of HO-1 with a TAT protein transduction domain (TAT/PTD), an 11-aa cell penetrating peptide from the human immunodeficiency virus TAT protein, into islets, improves islet viability in culture. HO-1 has been also shown to prevent *β*-cell apoptosis via p38 MAPK activation and the NF-*κ*B pathway in this study [37]. 

In addition to the *in vitro* experiments described above, Pileggi et al. showed that induction of HO-1 pharmacologically with cobalt protoporphyrin (CoPP) in recipients results in improved islet function in a marginal mass islet transplantation model in rodents, that is, fewer islets are required to achieve normoglycemia when transplanted into a syngeneic recipient that have been rendered diabetic by streptozotocin (STZ) treatment [36]. In addition, a short course of CoPP administration to recipients leads to long-term survival of DBA/2 (H-2^d^) islets in 30% of diabetic C57BL/6 (H-2^b^) recipients [38]. Most importantly, tolerance to transplanted islets is achieved as long-term graft-bearing animals rejected third-party islets while accepting a second-set donor-specific graft permanently, without additional treatment. It seems that induction of HO-1 leads to a donor-specific hyporesponsiveness in the CoPP-treated animals. Additionally, there is greatly reduced class II expression and a transient and powerful immunosuppression observed with reduced lymphocyte proliferative responses and increased proportions of T regulatory cells with decreased mononuclear cell infiltration into the graft [38, 39]. 

Another critical finding in the Pileggi study is that preconditioning of islets with hemin to induce HO-1 activity leads to improved graft survival in untreated recipients. Moreover, islet preconditioning provides additional advantages in HO-1-induced recipients that results in an increased proportion of long-term survival of transplanted islet allografts in recipients. Encouraged by this study, we tested whether HO-1 induction, or CO administration, to the islet donor, would sustain survival and function of transplanted islet allografts. Such an approach would avoid the toxicity associated with recipient treatment. Our data showed that without any other treatment, induction of HO-1 (20 mg/kg CoPP, 24 hr before isolation) or administering CO (250 ppm for 1 hr) only to the donor leads to long-term survival of DBA/2 (H-2^d^) islets in diabetic B6AF1 (H-2^b,k/d^) recipients, which are then antigen specifically tolerant. In essence, by using CO, we were mimicking the effects of HO-1 itself with one of its products. Several proinflammatory and proapoptotic genes that are strongly induced in islets after transplantation in the untreated situation were significantly suppressed after administering CO to the donor. These included TNF-*α*, inducible nitric oxide synthase (iNOS), monocyte chemoattractant protein-1 (MCP-1), granzyme B, and Fas/Fas ligand, all of which contribute to the pathogenesis and rejection of transplanted islets. Moreover, donor treatment is correlated with less infiltration of recipient macrophages into the transplanted islets [40]. We tested further whether CO conferred protection by suppressing Toll-like receptor 4 (TLR4) upregulation in pancreatic *β* cells. TLR4 is normally activated in islets during the isolation procedure, and its activation allows initiation of inflammation, which leads to islet allograft rejection. Donor treatment with CO suppresses TLR4 expression in freshly isolated islets as well as in transplanted islets at various times after transplantation. Islet allografts from TLR4-deficient mice survive indefinitely in BALB/c recipients and show significantly less inflammation after transplantation compared with grafts from a control donor. Isolated islets preinfected with a TLR4 dominant negative mutant virus before transplantation demonstrated prolonged survival in recipients. Despite the salutary effects of TLR4 suppression, HO-1 expression is still needed in the recipient for islet survival: TLR4-deficient islets were rejected promptly after being transplanted into recipients in which HO-1 activity was blocked [41]. Our data suggest that TLR4 induction in *β* cells is involved in *β* cell death and graft rejection after transplantation. CO exposure protects islets from rejection in part by blocking TLR4 upregulation in *β* cells. 

There are at several mechanisms by which HO-1 functions in the islet allogeneic transplantation model. First, HO-1 induction leads to a decreased inflammatory response in transplanted islets as compared to islets harvested from untreated donors. Inflammation not only contributes to *β* apoptosis but also heightens the alloaggressive immune response; thus, suppression of inflammation can lead to fewer cell deaths and a lesser immune rejection response. Second, the diminution of free radicals by HO-1 and its products should impart salutary effects as islet cells express lower levels of antioxidant genes than most other tissues of the body and are extremely sensitive to oxidative damages [42]. Third, HO-1 induction in the recipient increases the number/function of T regulatory cells, which generate a favorable microenvironment to transplanted islets and eventually contribute to the survival of those islets. Last but not least, HO-1 induction leads to the generation of biliverdin/bilirubin, requisite activation of BVR, and generation of CO, which can amplify the protective effects of HO-1 as CO is anti-inflammatory and antiapoptotic and the bile pigments are strong antioxidants and BVR can function to quell the inflammatory response [42]. Emerging data clearly demonstrate that BVR can regulate the inflammatory response through distinct intracellular signaling activity leading to increases in the anti-inflammatory cytokine IL-10 [43].

## 4. Overexpression of HO-1 Improves Function of an Islet Xenograft

The success of islet allogeneic transplantation is limited by the number of organ donors. Xenogenic donors (e.g., pig) offer potential unlimited sources of islets, and islet xenotransplantation is an alternative option for patients with T1D. However, despite a number of widely recognized advantages, the clinical application of porcine islet xenotransplantation has been hindered by a potent recipient xenospecific immune response and by the lack of a tolerable immunosuppressive strategy to overcome this barrier since cellular immune responses to xenogeneic cells are less clear and poorly understood [44–46]. A key for immunologic rejection in xenotransplantation is the damage to the graft due to chemotactic movement and infiltration of leukocytes into the graft [47]. The protective effects of HO-1 in the islet xenotransplantation model have been investigated by several groups, and improved survival and function of islet xenograft was observed when HO-1 was induced. For example, in a rat to mouse islet transplantation model, incubation of rat islets with CoPP before transplantation leads to a much better glucose-induced insulin secretion, longer graft survival time (14.63 ± 1.19 day versus 9.88 ± 2.17 days in control group), and less lymphocyte infiltration into the graft [48]. These results were confirmed by another group in which HO-1 was induced in male Sprague Dawley donor rats before islet isolation and transplanting the islets into C57BL/6 mice rendered diabetic by streptozotocin. Again, improved graft survival was observed [49]. In both studies, less lymphocyte infiltration and elevated IL-10 expression were observed in HO-1-induced islet xenografts, a phenomenon also observed in other models of HO-1 action (e.g., HO-1 increases expression of IL-10). IL-10 functions as a negative immunomodulatory factor and participates actively during inflammation, tumor immune responses, and the transplantation immune response. It promotes activation and differentiation of B cells, mediates humoral immunity, and inhibits proinflammatory cytokine expression and mononuclear cell expression of MHC II molecules and costimulatory molecules, as well as cytokine synthesis [50, 51]. Many of the observed protective effects of HO-1 in the xenotransplantation model might be mediated by IL-10 as HO-1 generates CO, which downregulated iNOS and upregulates IL-10 [15] and leads to protection to *β* cells. 

## 5. HO-1 Induction Interferes with the Onset of Diabetes

The pathophysiology of T1D is characterized by dysfunction and death of insulin-producing *β* cells in the pancreatic islets of Langerhans. At an early stage of disease onset, progressive mononuclear cells invade the islets and cause insulitis, a process that lasts for several weeks to months before severe *β* cell destruction occurs [52]. Mononuclear cell infiltration leads to the generation of reactive oxygen species (ROS) and proinflammatory cytokines including IL-1*β*, INF-*γ*, and TNF-*α* in pancreatic *β* cells. Elevated intracellular levels of ROS, including superoxide, hydrogen peroxide, and nitric oxide, leads to apoptotic and necrosis of *β* cells [53]. Increased proinflammatory cytokines contribute to insulitis [54]. The autoimmune nonobese diabetic (NOD) mouse resulting from autoreactive T-cell-mediated destruction of *β* cells is a useful and powerful model by which to study the development of T1D due to its similarity to human disease. Similar to human diabetes, the NOD mice develop insulitis that has been linked to activated macrophages and T cells the secretion of soluble mediators, such as oxygen radicals, NO, and cytokines [55]. HO-1 has been shown to slow progression to overt diabetes and interdict disease progress. Li and colleagues reported that HO-1 induction in NOD mice by weekly injection of CoPP reduces hyperglycemia and preserves the number of *β* cells via suppressing infiltration of CD11c^+^ cells. Increased phosphorylation of AKT, BcL-XL, and RSK levels and decreases in superoxide and 3-NT levels were observed in mice where HO-1 was induced [56]. The effect of HO-1 in preventing progression of overt diabetes was confirmed by another study in which HO-1 was induced in female NOD mice at 9 weeks of age with a single intravenous injection of a recombinant adeno-associated virus bearing the HO-1 gene (AAV-HO-1, 0.5 × 10^10^ – 2.5 × 10^10^ viruses/mouse). HO-1 induction significantly reduced destructive insulitis and the incidence of overt diabetes examined over a 15-week period. HO-1-mediated protection was associated with a lower type 1 T-helper-cell-mediated response. Adaptive transfer experiments into NOD-scid mice demonstrated that splenocytes isolated from AAV-HO-1 treated mice were less diabetogenic. However, no differences in CD4^+^CD25^+^ T regulatory cell infiltrates between saline-treated and the AAV-HO-1 treated group was observed [57]. In both studies discussed above, the protective effects of HO-1 could be substituted for with bilirubin and/or CO. 

Huang et al. confirmed the protective effect of HO-1 in preventing the onset of diabetes by generation of transgenic NOD mice in which the HO-1 transgene was driven by an insulin promoter (Plns-mHO-1) [58]. Although the overall expression level of HO-1 in transgenic islets was lower than that in nontransgenic islets stimulated with CoPP, a dramatic difference in insulitis and a lower incidence of diabetes were observed in the transgenic mice. Onset of diabetes was significantly delayed in the mHO-1-transgenic NOD mice, that is, spontaneous diabetes developed after 15 weeks of age in the mHO-1-transgenic NOD mouse compared to 12 weeks in age-matched controls. Diabetic incidence at 30 weeks of age was also significantly reduced (33.3% in the transgenic mice compared to 66.7% in controls). Moreover, islets from transgenic mice survived significantly longer than those harvested from wild-type donors when transplanted into new onset spontaneous diabetic female NOD recipients, although permanent protection from recurrence of diabetes was not achieved in this model [58]. The mHO-1-transgenic NOD islet grafts still expressed HO-1 at day 8 after transplantation. Preservation of islet architecture and intact insulin-secreting islets were observed within the pancreas [58]. However, local expression of HO-1 did not alter systemic or local lymphocyte and dendritic cell development in NOD mice which was in contrast to studies by another group where HO-1 was shown to inhibit maturation of dendritic cells and regulate the function of Th1 and Treg cells [59]. Conclusions from these studies were that the anti-inflammatory and antioxidant properties of HO-1 and its products interfered with the onset of diabetes in NOD mice.

## 6. HO-1 Induction Increases Insulin Sensitivity

The islets of Langerhans are equipped with a HO-CO pathway which constitutes a regulatory system of physiologic importance for the stimulation of insulin and glucagon release [60]. HO-1 expression and activity are reduced in patients with T2D compared to healthy individuals [61]. Overexpression of HO-1 activates the insulin-signaling pathway and has been shown to have unique and long-lasting antidiabetic effects in the rodent model of insulin resistance [62–64]. Moreover, HO-1 attenuates the oxidative destruction of adiponectin/insulin and improves insulin sensitivity and glucose metabolism in the STZ-induced T1D mouse model [65]. Induction of HO-1 by hemin increases plasma insulin level and enhances insulin sensitivity and improves glucose tolerance. The antidiabetic effects of hemin lasted for 2 months after termination of therapy and were accompanied by enhanced HO-1 expression and HO-1 activity of the soleus muscle, along with potentiation of plasma antioxidants including bilirubin, ferritin, and superoxide dismutase with elevation of the total antioxidant capacity. Hemin blocked C-Jun NH2-terminal kinase (JNK), a substance known to inhibit insulin biosynthesis, and suppressed markers/mediators of oxidative stress including 8-isoprostane, NF-*κ*B, and activating protein (AP-1 and AP-2) in the soleus muscle. In addition, hemin therapy significantly attenuated pancreatic histopathological lesions including acinar cell necrosis, interstitial edema, vacuolization, fibrosis, and mononuclear cell infiltration [66]. Thus, it seems that hemin-induced HO-1 can enhance the function of *β* cells via increase insulin sensitivity in the insulin resistance mouse model. 

HO-1 and its products are also protective against diabetes-related complications. Human HO-1 cDNA transferred into diabetic rats restored mitochondrial ADP/ATP and deoxynucleotide carriers [67]. Elevated HO-1 was associated with a significant increase in the phosphorylation of AKT and levels of Bcl-XL proteins. The cytoprotective mechanisms of HO-1 against oxidative stress involve an increase in the number of macrophages and antiapoptotic proteins as well as cytochrome c oxidase activity in this model [67]. Moreover, exogenous administration of the CO releasing molecule-3 (CORM)-3 and bilirubin prevents endothelial cell sloughing in diabetic rats, likely via a decrease in oxidative stress which represents a novel approach to prophylactic vascular protection in diabetics [64, 67, 68]. In addition to functioning as a positive modulator of glucose-stimulated insulin release, CO increases the propagation of Ca^2+^ signals with coordinating effects on the *β* cell rhythmicity [69]. 

## 7. A20 and Islet Survival and Function

A20, also known as the TNF-*α*-induced protein 3 (TNFAIP3), is a zinc-ring finger protein that was first identified as a cytokine-induced gene in human umbilical vein endothelial cells [70]. As a negative regulator of nuclear factor kappa B (NF-*κ*B) activation, A20 is recognized as a central and ubiquitous regulator of inflammation and as a potent antiapoptotic gene in certain cell types, including *β* cells [71–73]. A20 offers a potential therapeutic target for the treatment of diseases where apoptosis and/or the inflammatory response constitute components of the pathophysiology; thus, it is an ideal cytoprotective gene therapy candidate for T1D [74]. Overexpression of A20 by means of adenovirus-mediated gene transfer protects islets from IL-1*β*/INF-*γ* and Fas-induced apoptosis [75–77]. Transplantation of a suboptimal number of islets overexpressing A20 resulted in a cure in a high percentage of recipients compared to control islets. A20-expressing islets preserved functional *β* cell mass and are protected from cell death. The cytoprotective effect of A20 against apoptosis correlates with and is dependent on the abrogation of cytokine-induced NO production due to transcriptional blockade of iNOS induction; these data demonstrate a dual antiapoptotic and antiinflammatory function for A20 in *β* cells. 

## 8. Biliverdin Reductase and Islet Protection

The breakdown of heme continues with biliverdin as it is rapidly converted to bilirubin by BVR. BVR has, in recent years, evolved into a complex enzyme with additional functions including signal transduction and transcription factor activity. We include it here as many of the effects of HO-1 might be attributed in part to the additional functions for BVR resulting from the presence of biliverdin. A direct link between BVR and HO-1 in oxidative stress was described by Miralem et al. showing an attenuated HO-1 response to superoxide anion and arsenite in cells where BVR expression had been silenced [78]. BVR is a unique enzyme because it has been categorized to possess numerous biological functions. The reductase activity leads to the protective effects shown by biliverdin/bilirubin in a variety of experimental models of organ transplantation, endotoxic shock, and vascular injury [24]. BVR also exhibits kinase activity and corresponding signal transduction and more recently nuclear targeting and ability to regulate gene expression and the inflammatory response. Indeed, recent studies by us show that BVR is present on the cell surface and in this location binds biliverdin and in the conversion to bilirubin, activates a signal cascade leading to activation of Akt that in turn increases IL-10 expression [43]. The rapid conversion of biliverdin into bilirubin by BVR likely explains the beneficial effects observed with exogenous biliverdin administration. Indeed the signalling and transcriptional activity of BVR in addition to generating increased bilirubin may act synergistically and explain the mechanism of biliverdin-induced protection. Modulation of BVR itself directly regulates the inflammatory response and, *in vivo*, can prevent acute liver injury [43].

In addition to its anti-inflammatory effects, BVR modulates glucose uptake and insulin resistance by decreasing glucose transport and metabolism in competition with the insulin receptor substrate-1 (IRS-1) for phosphorylation by insulin receptor kinase (IRK). This leads to a reduced binding with PI3 K and accelerated degradation of IRS [79]. Therefore, therapeutic molecules designed to suppress the kinase activity of BVR may play an important role in the reversal of diabetes. 

As previously discussed, islet allografts suffer a gradual loss of function in response to oxidative stress, inflammation, and apoptosis as well as activation of the humoral immune response. Although intraportal infusion represents the most frequent procedure in the clinic for human islet transplant, a high percentage of islets are destroyed at a very early posttransplant stage because of the instant blood-mediated inflammatory response [80, 81]. Bilirubin administration reduced apoptosis and improved insulin secretion in an *in vitro* model in INS-1 cells when challenged with nonspecific inflammation induced by cytokines. Moreover, bilirubin administration led to improved glucose control and protection of islets grafts in a syngeneic rat model of intraportal islet transplantation by inhibiting the production of IL-1*β*, TNF-*α*, ICAM-1, and MCP-1, as well as infiltration of Kupffer cells [82].

Bilirubin administration to the donor, and even more so to cultured islets, without further treatment of the recipient would represent a great advantage in clinical practice. Freshly isolated islets from bilirubin-treated donors led to a strong expression of the protective genes HO-1 and bcl-2 and a clear suppression of the proapoptotic and proinflammatory genes caspase-3, caspase-8, and MCP-1 [83, 84]. This protective effect of bilirubin leads to reduced *β*-cell destruction after transplantation, reduced macrophages infiltration, and decreased expression of MCP-1, BID, caspase-3, -8, and -9, TNF-*α*, iNOS, Fas, TRAIL-R, and CXCL10 in the graft after allogeneic transplantation [84]. The therapeutic potential of bilirubin is further corroborated by data reported in Gunn rats (genetically predisposed to high bilirubin levels) rendered diabetic by streptozotocin administration in which the typical hyperbilirubinemia represents a “natural” protection to oxidative stress [85]. 

Bilirubin administration to recipients clearly improves graft survival by inducing immune tolerance via *de novo* generation of T regulatory cells. Bilirubin was no longer protective when CD4^+^CD25^+^ Treg cells were depleted from recipients prior to transplantation suggesting that Tregs were critical in the ability of bilirubin to protect [86]. Moreover, as previously shown in kidney and heart transplantation models, dual therapy by combining CO and biliverdin enhanced long-term graft survival [87]. Interestingly, a recent study in a rodent model of type 2 diabetes describes the protective effects of biliverdin administered orally [88]. Biliverdin inhibited *β*-cell injury caused by oxidative stress and resulted in glucose tolerance and improved function. Biliverdin has been shown to increase the insulin content, reduce Bax, and enhance Pdx1 expression in diabetic mice compared to control [88]. Similar effects in T1D models which would be a significant turning point for potential clinical use have not yet been tested.

## 9. Other Protective Genes/Factors that Can Increase Islet Survival and Function

There are many other protective genes that have been shown to protect pancreatic *β* cells. Mancarella et al. reported that exposing human islets to the nonpeptidyl low molecular weight radical scavenger IAC [bis(1-hydroxy-2,2,6,6-tetramethyl-4-piperidiny) decanedioate dihydrochloride] on isolated human islet cells protected them from isolation and culture-induced oxidative stress [89]. Enhancing expression of suppressor of cytokine signaling 1 (SOCS1) in isolated rat islets prior to transplantation protected them from apoptotic loss and prolonged survival [90]. Transduction of NOD islets with the antioxidative gene thioredoxin (TRX, reactive oxygen species scavenger and antiapoptotic) using a lentiviral vector before transplantation prolonged islet graft survival in NOD mice [91]. Anthocyanins from Chinese Bayberry protects *β* cells against hydrogen-peroxide-induced necrosis and apoptosis via upregulation of HO-1 [92]. Adenoviral transfection of human islets with human X-linked inhibitor of apoptosis provided protection from inflammatory cytokines and improved their viability and function [2, 93–104]. 

## 10. Conclusion

Due to the complex nature of the pathogenesis of diabetes, interfering with antigenic recognition and/or cell death, imparting tolerance, immunoregulation, and cell protection offer a promising form of immunotherapy [3, 105]. Based on the potent cytoprotective and immunoregulatory effects of HO-1, A20, BVR, and other protective genes, targeting strategies aimed to induce their expression or by administering one or more of their products hold great promise in protecting islet cells from apoptosis and may prove critical as potential therapies for diabetes and other human diseases.

## Abbreviations

T1D: Type 1 diabetes
HO-1: Heme oxygenase-1
BVR: Biliverdin reductase
IL-1*β*: Interleukin-1*β*
CoPP: Cobalt protoporphyrin
NOD: Nonobese diabetic
STZ: Streptozotocin
CORM: CO releasing molecule
TLR4: Toll-like receptor 4
iNOS: Inducible nitric oxide synthase
MCP-1: Monocyte chemoattractant protein-1.
